# Women’s Sexual Dysfunctions Following Stem Cell Transplant and the Impact on Couple Relationship

**DOI:** 10.3390/life14010035

**Published:** 2023-12-25

**Authors:** Mihaela Plotogea, Anca Zgura, Claudia Mehedințu, Francesca Scurtu, Aida Petca, Valentin Nicolae Varlas, Roxana Georgiana Bors, Antoine Edu, Oana-Maria Ionescu, Mihaela Andreescu, Radu Nicolae Mateescu, Al Jashi Isam

**Affiliations:** 1 Department of Obstetrics and Gynecology, “Nicolae Malaxa” Clinical Hospital, 022441 Bucharest, Romania; 2 Department of Obstetrics and Gynecology, “Carol Davila” University of Medicine and Pharmacy, 020021 Bucharest, Romania; 3 Department of Obstetrics and Gynecology, “Filantropia” Clinical Hospital, 011179 Bucharest, Romania; 4 Faculty of Medicine, “Titu Maiorescu” University, 031593 Bucharest, Romania

**Keywords:** hematological malignancy, female sexual function index, bone marrow transplant, premature ovarian failure, vulvovaginal atrophy, sexual dysfunction

## Abstract

Stem cell transplant proved its efficacy in increasing the survival rate among young patients diagnosed with hematological malignancies. A transplant conditioning regimen is particularly destructive on the genital system, often determining premature ovarian failure, accompanied by vulvovaginal atrophy and sexual dysfunctions. The aims of the present study were, first, to evaluate sexual dysfunctions among transplanted women, using clinical examination and the female sexual function index (FSFI), and second, to determine their impact on a couple’s relationship. A prospective observational comparative study was performed and included 38 patients who underwent allogenic stem cell transplant (SCT) procedures for different hematological malignancies and 38 healthy patients (control group). This study included baseline evaluation, one-year, and three-year follow-up visits. In addition to anamnesis and medically obtained information, FSFI was evaluated to determine the impact of gynecological damage in a subjective manner. In the study group, vulvovaginal atrophy was diagnosed in 76.32%, with subsequent sexual dysfunctions in 92.10% of patients, based on FSFI scoring. Even though the results improved throughout the study, at the last visit, mild vulvovaginal atrophy was diagnosed in 81.58% of patients, and the FSFI score was abnormal for 21.05%. When compared to the control group, both sexual dysfunctions and FSFI results were considerably impaired, with statistical significance. There is a confirmed negative impact of sexual dysfunctions and self-declared FSFI on couple/marital status and couple relationships, with statistical significance, at the last visit. In conclusion, anatomical, functional, and psychological difficulties are a reality of long-term survivors after a stem cell transplant. They should be addressed and assessed equally to other medical conditions, as they may determine serious consequences and impact the sexual quality of life and the couple’s relationship.

## 1. Introduction

An increasing number of hematological malignancies have been reported worldwide among young, reproductive-age women, both as a primary and secondary disease [1]. Frequently, the treatment consolidation requires an allogeneic stem cell transplant procedure, whose regimen includes aggressive gonadotoxic chemotherapy and, sometimes, total body irradiation [2,3]. Although it increases patients’ survival rate and improves life expectancy, various short- and long-term consequences of the treatment may appear. The damaging effects extend beyond targeted cells and may involve different organs and systems [4].

The reproductive system is often seriously damaged, both functionally and as concerns of endocrine function, namely hormone secretion and reproductive status [5]. The impact of gonadotoxic chemotherapy (as included in the transplant regimen), associated or not with total body irradiation (TBI), is highly dependent on several variables. Firstly, age at hematological diagnosis is directly proportionate with an ovarian reserve and may determine if a patient will have a residual ovarian function or not [6]. Also, the type of hematological malignancy, type of bone marrow transplant (auto or allogenic), the treatment conditioning, and the association of TBI will determine the extent of anatomical and endocrine damage [3]. Endocrine abnormalities reflect, in a temporary or permanent ovarian insufficiency, the type of ovarian damage, being time- and dose-dependent on chemotherapy, associated or not without irradiation [7]. Frequently, those anomalies are accompanied by genitourinary syndrome. The prevalence of premature ovarian failure is reported differently among various researchers, as in allogenic transplantation, which includes aggressive gonadotoxic medication [8]. It can be diagnosed in up to 72.7% of cases [6] and in more than 90% when TBI is associated [9,10].

As regards anatomical, functional, and sexual alterations, the severity is proportionate with ovarian damage, as symptoms may decrease if the patient restores its endocrine function and women start experiencing spontaneous menstruations. If the ovarian reserve is destroyed, the patient will be further diagnosed with premature ovarian failure and genitourinary syndrome of menopause, with severe vulvovaginal atrophy and sexual dysfunctions, with treatment requirement [11]. Although most patients experience both anatomical and sexual discomfort following the transplant procedure, is it highly important to evaluate and determine the extent of gynecological anomalies and to prescribe appropriate treatment to restore the sexual function of involved patients [12]. Our study aims to determine the severity and the incidence of mentioned alterations, as well as to evaluate the impact of sexual dysfunctions, using the female sexual function index (FSFI), on women’s sexual life and couple relationships [13,14].

## 2. Materials and Methods

The present research is a prospective and observational comparative study conducted between 2014 and 2020 and took place within the Department of Obstetrics and Gynecology ‘Nicolae Malaxa’ Clinical Hospital, with the aid of the Department of Bone Marrow Transplant ‘Fundeni’ Clinical Institute in Bucharest, Romania. The presented study evaluates sexual dysfunctions and related complications of young women following stem cell transplantation. It is part of a more extensive study that evaluated young women who underwent bone marrow transplantation procedures and further determined gynecological-related complications, as well as the psychological impact of the procedure. The patients’ inclusion, evaluation, treatment, follow-up, and statistical analysis proceeded with the approval of the Local Ethics Committee.

Two patient groups were compared and studied. The study group included 38 women diagnosed with hematological malignancy who underwent an allogeneic stem cell transplant, in complete remission from the initial malignancy, with ages between 20 and 35 years, and they completed all three visits in the study design. Transplant procedure was referred for patients for acute lymphoblastic leukemia (36.8%), acute myeloblastic leukemia (28.9%), chronic myeloblastic leukemia (5.3%), Hodgkin lymphoma (23.7%), and non-Hodgkin’s lymphoma (5.3%). Patients underwent chemotherapy as a part of the hematological malignancy, considered differently as diagnostic protocol, as well as transplant regimen, also personalized and accordingly to diagnosis, general health condition, genetic mutations, and residual disease. For all patients, the administration of aggressive and gonadotoxic chemotherapy agents was recorded, especially alkylating agents known for their ovarian-damaging effect.

All patients included in the study group were one year after the transplant procedure. It was considered that after at least one year following the transplant procedure, patients would be free of post-transplant medication, and ovarian function might be restored, in case of residual ovarian reserve. Women with major complications, vital organ insufficiencies, uncontrolled acute or chronic graft versus host disease, secondary malignancy, and premature ovarian failure before initial diagnosis were not included in the study. A control group was added to accurately assess patients by comparing data to a healthy population. The control group included 38 healthy women, aged 20 to 35 years, using the age, living habitats, and level of education criteria, and excluded women diagnosed with premature ovarian failure to properly compare and examine data obtained from the study group.

Data were collected at baseline, one year after, and three years following the first visit. The initial visit recorded general information for both groups regarding age, body mass index, education, living habitats (rural/urban), couple/marital status, medical, obstetrical, and gynecological history, spontaneous menstruations, and gynecological and paraclinical evaluation. For the study group, baseline information also recorded specific data related to the hematological malignancy and treatment protocol, including the total body irradiation procedure. Following baseline visit gynecological evaluation, patients with abnormal findings, both clinically and paraclinical, namely 29 women out of the total of 38, were considered for systemic hormonal substitution, associated or not with local hormonal administration, if there were no contraindications regarding initial malignancy, transplant procedure, pot-transplant treatment, or related complications, and with patient’s consent. The treatment was recommended as ovarian insufficiency protocol, and not in a randomized pattern, namely systemic estrogen, progesterone therapy, and local dehydroepiandrosterone.

The second and third visits included information, such as couple/marital status, gynecological examination, and paraclinical evaluation. At all visits, patients completed the female sexual function index (FSFI), which includes questions about sexuality and couples’ relationships. The gynecological examination focused on signs of vulvar and vaginal atrophy, as part of the genitourinary syndrome of menopause that is frequently diagnosed in patients after stem cell transplant, which also presents as temporary or permanent induced ovarian insufficiency. Objective-provided information from the clinical exam was completed with subjective data using the FSFI questionnaire that was addressed to the patients to collect information regarding sexual function and couple relationships. Although the initial scale was designed to evaluate sexuality during the previous four weeks, at baseline, patients completed for the previous year (time passed since transplant procedure), and at visits two and three, they answered the questions for the time that passed since the last visit. The FSFI is a multidimensional, self-reporting, 19-item questionnaire developed and validated to evaluate sexual function in women. It includes six domains and assesses several sexual functions, namely desire for sexual intercourse, arousal during sexual activity, lubrication, satisfaction, and orgasm concerning sexual relations, as well as pain during and after sexual intercourse. Data regarding the couple’s intimate relations and quality of life related to sexual dysfunctions were also collected using the above-mentioned questionnaire. Domain desire, which includes questions 1 and 2, has a range of scores per question from 1 to 5 and a factor of 0.6. The final domain score ranges from 1.2 to 6. Arousal, indicated by questions 3 to 6, and lubrication, questions 7 to 10, have a range of scores from 0 to 5, a factor of 0.3, and a final domain score between 0 and 6. Domains of orgasm, satisfaction, and pain, including questions 11 to 13, 14 to 16, and 17 to 19 have a range score from 0 to 5, a domain factor of 0.4, and a final score between 0 and 6. The analysis of the data collected and the final score were performed as recommended by the validated scale. The final score varies between 1.2 and 36.0, and a result below a score of 26.55 is classified as sexual dysfunction.

### Statistical Analysis

All data were initially collected with Microsoft Excel (Version 16.0). For statistical analysis, we used specialized software IBM SPSS Statistics, version 26 (IBM Corp., Armonk, NY, USA). Descriptive analysis was performed to express demographic and clinical variables, age, couple/marital status, total body irradiation, presence of spontaneous menstruations (as a sign of residual ovarian function), vulvovaginal atrophy, and FSFI. Means ± standard deviations and ranges or medians and ranges were used for continuous variables. Categorical variables were expressed as frequencies/absolute numbers with percentages. Group differences were tested with a Chi-square test and one-way ANOVA, whichever was relevant. Paired samples *t*-test was used to assess the evolution of a continuous outcome (ex. FSFI) across time or within-subjects across two observations. *p*-values less than 0.05 (95% CI) were considered statistically significant, thus rejecting the null hypothesis.

## 3. Results

The present study enrolled 38 patients, aged 20 to 35, according to inclusion and exclusion criteria. A control group of 38 women was also included, according to the mentioned criteria. Anamnestic characteristics were documented at baseline, completed by paraclinical measurements and clinical examination. Table 1 below presents relevant data for the study. Considered important to determining gynecological damage, the total body irradiation procedure was added to the data presented at baseline. As may be observed, at the first visit, only nine patients had restored spontaneous menstruations, especially younger patients, and no women with age above 29. Also, no patient that had TBI included in the transplant regimen (three patients), despite age, had restored menstruation.

**Figure 1:**
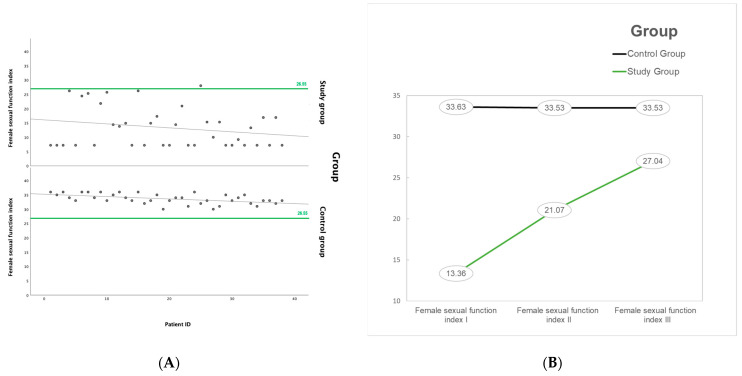
(**A**,**B**) Female sexual function index of study and control group at baseline: progression from first to last visit.

Both vulvovaginal atrophy and the sexual function index were severely impaired at baseline. Severe atrophy was clinically diagnosed in 50% of patients and moderate form in 26.32%. Accumulated, serious damage of external genitalia is observed in 76.32% of women. A normal examination may be seen in patients that restored spontaneous menstruation. In addition, the data collected from the female sexual function index scale confirmed the clinical anomalies. The median score in the study group was 13.36, but only one patient had a result above 26.55 (results below considered to define sexual dysfunctions), and in two patients, the final score was 26. All patients from the control group had a normal result on the FSFI scale, considered to be above 26.55, with no sexual dysfunction being diagnosed inside the group. When compared, the differences between the study group and the healthy population were statistically significant, as seen in Figure 1 below. Moreover, all patients who underwent a TBI procedure—patients 1, 5, and 30—reported low FSFI scores at all visits, despite the continuous systemic and intravaginal treatment.

In addition, paraclinical examination revealed ultrasound anomalies, such as reduced antral follicle count (AFC) in 36.8%, and no follicle visible for 63.2% of control group patients, while no patient had a normal ovarian aspect. In the control group, only 3.95% of patients showed reduced AFC, while the rest had a normal ultrasound aspect. Those alterations from the study group were sustained by ovarian biomarker evaluation. Increased follicle-stimulating hormone (FSH) and luteinizing hormone serum values were recorded in 76.31% of cases, with low levels of estradiol accordingly. The hormonal levels were also recorded at following visits, with abnormal results and menopausal values for patients who were not administrated systemic hormone substitution. As for the rest, due to hormone intake, the values were influenced by the treatment, and the evaluation for ovarian function was assessed using AMH serum values. No abnormal findings were recorded in the control group.

Anti-Mullerian hormone was particularly assessed and compared between groups at all three visits, as can be observed in Table 2. The AMH mean value was found to be considerably low in the study group throughout the study, from the initial to the last visit. Based on both paraclinical values performed at all three visits, 29 patients, 76.32%, were diagnosed with premature ovarian failure, with a recorded AMH serum value <0.01 ng/mL. For the rest of 23.68%, the ovarian reserve was found to be very low, and patients were particularly medically advised regarding their fertility status. Following paraclinical assessments revealed that the serum AMH value was constantly decreasing, with a negative value, namely <0.01 ng/mL for the 29 patients diagnosed with POF, respectively, in continuous decline for the remaining 9 patients with residual endocrine function. The decrease in the serum AMH value was evaluated throughout the study. The maximum value was found to be 1.6 ng/mL at the initial visit and 1.2 ng/mL at the last visit, significantly reduced compared to the control group. A decline in the medium serum AMH value was observed for the nine patients with residual function in the study group, which dropped from 1.32 ng/mL to 0.87 ng/mL at the last visit. When compared between groups, the serum AMH values, namely maximum, medium, as well as progression throughout the study, as detailed in Table 2., were found to have statistically significant differences, with a particular focus on patients with ovarian endocrine function, from both groups.

After the initial visit, patients in the study group were recommended systemic hormonal substitution and vaginal dehydroepiandrosterone (DHEA), if there were no contraindications related to other medication, concurrent medical conditions, or if a patient refused. As may be observed in Table 3A–D below, both the genital atrophy and FSFI result improved at the following visits; at visit 3, with a high correlation, Cohen’s effect was >0.8.

Anomalies in FSFI results were seen even for patients with residual ovarian function and in the absence of vulvovaginal atrophy at the first gynecological examination. At the last visit, the clinical exam only revealed minor anomalies, with results of the FSFI scale completed by patients presented below. Table 4A highlights the major statistically relevant differences (*p* < 0.001) in the FSFI result of the study group at all visits, when compared to the normal population. Both variables, namely FSFI and vulvovaginal atrophy, showed improved results. There is a constant increase in FSFI mean results, especially when DHEA is associated with systemic therapy. In Table 4B, the correlation between FSFI results and the severity of vulvovaginal atrophy can be noticed at each of the three visits performed, with statistical significance at both the first and last visit (*p* < 0.001) and p equal to 0.001 at the second visit. This study also confirms a strong correlation between the improvement in the two variables discussed above with systemic and, especially, local treatment. Table 4C,D show a significant correlation between both vulvovaginal anomalies and FSFI results with local DHEA treatment, with a *p*-value < 0.001 at the last visit.

**Figure 2:**
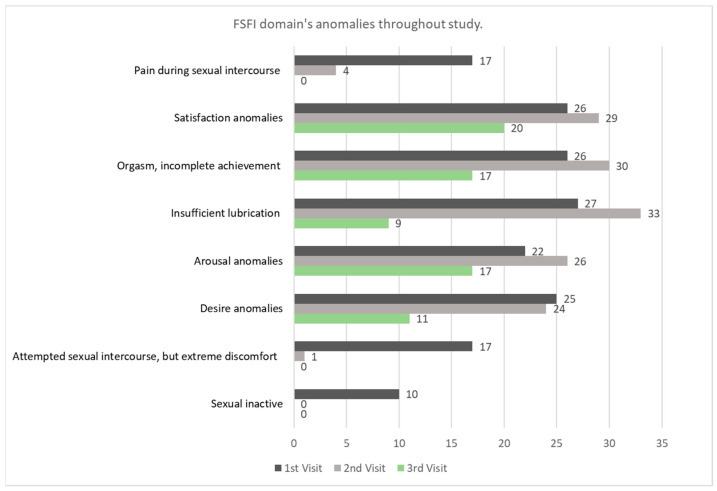
FSFI domain’s anomalies of study group throughout study.

In addition to the final FSFI scores of patients from the study group, we performed a detailed analysis of each domain included in the female sexual function. In Figure 2, we observe that 10 patients were sexually inactive at the first visit, while the other 17 experienced severe discomfort when attempting sexual intercourse. Also, women described anomalies in most FSFI domains, despite proper treatment. Pain during and after sexual relations improved throughout the study, but psychological issues are still recalled years following the transplant procedure. There are concerns about domains, like desire, arousal, orgasm, and satisfaction, detailed below at all visits. In the last visit four years following the transplant procedure, 28.94% of patients recalled desire anomalies, 44.73% arousal issues, 23.68% insufficient lubrication, 44.73% difficulties from achieving orgasms, and 52.63% decreased satisfaction regarding sexuality and intimacy. At detailed anamnesis, patients experienced lowered self-esteem, related not only to sexual dysfunctions and intimacy satisfaction but also to premature ovarian failure. The overall impact of the sexual quality of life and premature ovarian failure (POF) plays a crucial role in the present and future personal and couple relationships.

Table 5A presents the results of couples’ relationships, with the differences at the initial visit compared between groups. The last visit highlights the significant differences, with a *p* < 0.001. Table 5B shows the impact of FSFI results on the patient’s relationship/marital status at the last visit (*p* < 0.001), confirming that a reduced FSFI and sexual dysfunctions may considerably contribute to alterations in the sexual life and couples’ relationship. Table 5C displays the impact of both sexual dysfunctions, confirmed by the FSFI scale, and couple/marital status, which applies to older patients, age group 28 or above. Younger patients were less affected, maybe because most patients are highly educated or in the process of education and postpone a serious relationship for a career.

As can be observed, there is a high incidence of anatomical and functional anomalies of the genital tract, with significant early vulvovaginal atrophy following stem cell transplant-associated treatment. Even if restored in most cases, at the last visit, four years after the SCT, minor alterations may still appear. Regarding the sexual quality of life and sexual dysfunctions, the FSFI result increased from an initial mean value of 13.36 to 21.07 and 27.04 at the last visit. Those results show constant alterations and impact on sexuality, and as may result from an impaired sexual function, a considerable decrease in self-esteem, negatively affected intimacy and couples’ relationships.

## 4. Discussion

Stem cell transplant has been confirmed as beneficial in consolidating the treatment of hematological malignancies. With promising results and increased survival rates, the procedure has extended indications in various malignant and non-malignant, primary, or secondary medical conditions [1,15]. In the present study, all patients underwent the transplant procedure due to several types of hematological cancer, namely acute lymphoblastic leukemia, acute and chronic myeloblastic leukemia, Hodgkin, and non-Hodgkin lymphoma. The transplant regimen includes aggressive chemotherapy, such as alkylating agents, and, in selected cases, total body irradiation [2,3]. The impact of chemotherapy and irradiation can determine detrimental effects that extend beyond targeted tissues and cells. Various organs and systems may be injured, some temporarily, others permanently [16].

The strength of our study is that this is the first study ever performed in Romania regarding the sexual dysfunctions of women with hematological malignancies following stem cell transplant and its impact on the couple’s relationship. Moreover, most of the studies concerning gynecological long-term consequences of stem cell transplant highlighted the ovarian endocrine function and, to a laser extent, the sexual dysfunction impact on the couple’s relationship. However, this study has its limitations, mainly due to the small number of patients enrolled. Nevertheless, the number of patients included in our study is not low for the analyzed pathology. Stem cell transplant is not an everyday procedure, and serious complications may develop following SCT, but the challenge came to enroll young, stable women, without important SCT consequences, willing to participate and evaluate sexual function after overcoming a life-threatening disease. Sexual function, intimacy, and social and personal life are rarely assessed and discussed with cancer survivors, without admitting that they may seriously impact recovery, reintegration, and improve patients’ quality of life. For a more profound analysis, a multicenter study will surely provide more information regarding the impact of hematological malignancy treatment on female sexual function and couples’ relationships.

The genital tract is commonly negatively impaired due to aggressive chemotherapeutical drugs and TBI. The ovarian tissue is highly sensitive to alkylating agents, widely used in hematological malignancies, and to pelvic irradiation, which causes significant damage [3,17,18]. With non-renewable characteristics and, in the case of continuous and prolonged chemotherapy administration, associated or not with TBI, the destruction of ovarian tissue may be permanent, with no ovarian reserve left intact, determining POF. Iatrogenic POF is frequently encountered among young patients who underwent an SCT procedure [19], as well as subsequent menopausal symptoms and signs, sexual dysfunctions, secondary infertility, and impaired quality of life [11,12]. The incidence of POF following SCT is reported differently by various studies, but the incidence may be as high as 90% or even greater [6,19,20]. The risk of POF is associated with age, as women over 30 years are at higher risk of ovarian failure, as well as hematological malignancy (the greatest for leukemia), chemotherapy drugs (type, accumulated dose), and TBI procedure included in the transplant regimen [8,21,22]. In our study, 76.32% of women were diagnosed with premature ovarian failure based on paraclinical evaluation and clinical signs at the initial visit and confirmed at the following, like the findings reported by Forgeard et al. [23]. At the last visit, the ovarian reserve was decreased for the other nine patients included in the study group (confirmed by anti-Mullerian hormone and antral follicle count on ultrasound). The decrease in ovarian reserve due to gonadotoxic chemotherapy was confirmed by various studies, and young women after cancer treatment are more likely to experience early menopause compared to the normal population [24,25].

As related to ovarian insufficiency, patients from our study group were diagnosed with severe subsequent signs and symptoms, both systemic and local. Aside from general symptoms characteristic of induced menopause, patients presented moderate and severe vulvovaginal atrophy at the first visit, accumulated in 76.32% of women. Several types of research related to gynecological anomalies, such as genitourinary syndrome of menopause, genital graft versus host disease, ovarian insufficiency, and so on, have been reported in the literature. The frequency of symptoms and signs is particularly related to age, transplant regimen, and post-treatment complications [26]. Despite systemic and specific DHEA vaginal treatment, for patients from the study group, alterations may still be observed years following the transplant procedure, and only mild anomalies were reported for 81.58% of patients.

Clinical anomalies were highly associated with patients’ declared sexual dysfunctions and FSFI scale results. One year following the SCT visit, we reported sexual dysfunctions in 92.10% of patients. Even for women with residual ovarian function and regained spontaneous menstruations without clinically observed vulvovaginal atrophy, the FSFI was decreased in eight out of the nine patients. The incidence of sexual dysfunctions in our study was above that reported by others one year following the transplant procedure. Noerskov et al. found anomalies for 60% of women [27]. At the follow-up visits, FSFI results were decreased by 68.42% and 21.05% of patients with statistical significance. Schover et al. and Sopfe et al. also reported sexual function anomalies in approximately 60% of female patients, although they included all cancers, and SCT was not specifically addressed [12,28]. Nevertheless, sexual concerns are more frequently observed following aggressive cancer treatment and vary between 30 and 100% of cancer survivors [12]. In our study, years after cancer treatment, and regardless of improvements in both genital atrophy and FSFI scores, women recall anomalies in the matter of frequency of sexual intercourse, lubrication, desire, satisfaction, and achieving orgasms. Those difficulties were reported by several other studies and confirmed to be statistically significant [29]. Although cancer treatment is associated with sexual dysfunctions, such long-term consequences are more likely to be diagnosed following SCT, known to determine an increased risk of premature ovarian failure and related decreased sexual quality of life [6,30]. As reported by Alsuliman et al., the sexual and emotional consequences of SCT are frequently encountered in surviving patients. They present in a complex manner, making it difficult to evaluate objectively. Medical personnel often poorly manage and assess, with negative impacts on patients’ quality of life [31]. Sexual quality of life may be impacted by a variety of factors, both physical and psychological, and frequently, the impact is bidirectional, especially on the patient’s recovery after the hematological cancer treatment [30]. The anatomical and functional anomalies may be restored partially or completely with proper treatment. In contrast, sexual satisfaction is often influenced by the psychological health of the individual. There is also the importance of specific medical care related to sexual dysfunctions, and researchers reported improved results when proper care is addressed, and sexual counseling is performed [32]. Following SCT, there is various and serious psychological distress, which additionally impacts sexual quality of life [33].

As regards the association of sexual dysfunctions and FSFI final score with couples’ relationships, our study displayed a significant negative impact both upon couple/marital status or steady, long-term relationships. At baseline, 73.68% of patients declared that they were single, and at the last visit, 63.15% were still unmarried or involved in a long-term relationship. This aspect also correlates with statistically significant FSFI scores and the impact of FSFI on the couple/marital status of studied patients. Data are supported by various research, which describes considerable concern regarding couple/marital status, difficulties in establishing new relationships, and anxiety related to couples’ breakdown or breakup [30,34]. The psychological impact of SCT’s long-term gynecological consequences may be related not only to sexual dysfunctions but also to infertility [7,35]. Patients included in our study reported increased concerns about future fertility, both diagnosed with POF and those with residual function, and this will significantly impact present and future relationships. Comorbidities and medical conditions that may arise as short- or long-term complications of SCT, together with all other physical and psychological alterations related to initial disease and aggressive treatment, are well known to significantly impact a patient’s well-being [36]. The decreased self-esteem associated with fertility issues and sexual dysfunctions, added to other possible transplant complications, will play a crucial role in the patient’s recovery, social reintegration, personal and intimate relationships, and overall quality of life [6,12,37].

## 5. Conclusions

A series of short- and long-term complications may be associated with stem cell transplant. Most frequently, gynecological consequences are diagnosed in survivor patients, such as ovarian insufficiency, permanent ovarian failure, sexual dysfunctions, and related complications. Our study described and analyzed anatomical anomalies from self-provided data using a sexual questionnaire, and significant alterations were confirmed in the study group when compared to the control group. Couple/marital status and long-term relationships were impaired among the study group, at all visits, but especially decreased at the last visit compared to the healthy population. The results also showed an increased correlation between declared sexual dysfunctions and couples’ relationships, namely couple/marital status, for patients who underwent a transplant procedure. The premature onset of menopause related to the depletion of ovarian reserve consecutive to transplant procedure leads to sexual and intimacy anomalies that may have a detrimental impact on survivors’ quality of life.

## Figures and Tables

**Table 1:** Baseline characteristics of study group.

Variable	Patients N = 38
Age, mean ± SD	27 ± 3.54
Total body irradiation, n (%)	
Yes	3 (7.89%)
No	35 (92.11%)
Spontaneous menstruations, n (%)	
Yes (residual ovarian function)	9 (23.68%)
No	29 (76.32%)
Vulvovaginal atrophy, n (%)	
Absent	9 (23.68%)
Mild	0 (0%)
Moderate	10 (26.32%)
Severe	19 (50%)
Female sexual function index, mean ± SD	13.36 ± 7.01

**Table 2:** Comparison of Anti-Mullerian serum value between the study group and control group throughout the study.

Descriptives									
		N	Mean	Std. Deviation	Std. Error	95% Confidence Interval for Mean		Minimum	Maximum
						Lower Bound	Upper Bound		
AMH serum value 1st visit	Study group	38	0.3600	0.56154	0.09109	0.1754	0.5446	0.01	1.60
	Control group	38	3.0868	1.04911	0.17019	2.7420	3.4317	0.90	5.10
	Total	76	1.7234	1.60694	0.18433	1.3562	2.0906	0.01	5.10
AMH serum value 2nd visit	Study group	38	0.2847	0.48828	0.07921	0.1242	0.4452	0.01	1.50
	Control group	38	2.9632	1.02335	0.16601	2.6268	3.2995	0.80	4.90
	Total	76	1.6239	1.56578	0.17961	1.2662	1.9817	0.01	4.90
AMH serum value 3rd visit	Study group	38	0.2155	0.40161	0.06515	0.0835	0.3475	0.01	1.20
	Control group	38	2.8158	0.98818	0.16030	2.4910	3.1406	0.70	4.70
	Patients with residual function in study group	9	0.8778	0.31535	0.10512	0.6354	1.1202	0.30	1.20
	Total	76	1.5157	1.50804	0.17298	1.1711	1.8603	0.01	4.70

**Table 3:** (**A**–**D**) Progression of FSFI and genital atrophy of study group throughout the study.

**(A)**					
**Variable**		**First Visit**	**Second Visit**	**Third Visit**	
Female sexual function index, mean ± SD		13.36 ± 7.01	21.07 ± 7.05	27.04 ± 3.81	
Vulvovaginal atrophy, n(%)					
Absent		9 (23.68%)	7 (18.42%)	7 (18.42%)	
Mild		0 (0%)	26 (68.42%)	31 (81.58%)	
Moderate		10 (26.32%)	4 (10.53%)	0 (0%)	
Severe		19 (50%)	1 (2.63%)	0 (0%)	
**(B)**					
**Pair**		***p***	**Effect Size Cohen’s d**	**Strength of Association (ω2)**	
Female sexual function index I & Female sexual function index II		<0.001	−1.91	0.65	
Female sexual function index II & Female sexual function index III		<0.001	−1.58	0.56	
Female sexual function index I & Female sexual function index III		<0.001	−2.89	0.81	
**(C)**					
**Vulvovaginal Atrophy I, n (%)**	**Vulvovaginal Atrophy II, n (%)**				
	**Absent**	**Mild**	**Moderate**	**Severe**	**Total**
Absent	7	2	0	0	9
Mild	0	0	0	0	0
Moderate	0	7	3	0	10
Severe	0	17	1	1	19
Total	7	26	4	1	38
**(D)**					
**Vulvovaginal Atrophy II, n (%)**	**Vulvovaginal Atrophy III, n (%)**				
	**Absent**	**Mild**	**Moderate**	**Severe**	**Total**
Absent	7	0	0	0	7
Mild	0	26	0	0	26
Moderate	0	4	0	0	4
Severe	0	1	0	0	1
Total	7	31	0	0	38

**Table 4:** (**A**–**D**) FSFI and vulvovaginal atrophy: progression in the study group and compared to the control group, association, and evolution with vaginal DHEA.

**(A)**						
**Variable**		**Study Group (N = 38)**		**Control Group (N = 38)**		***p*** **(95% CI)**
Female sexual function index I		13.36 ± 7.01		33.63 ± 1.77		<0.001
Female sexual function index II		21.07 ± 7.04		33.53 ± 1.50		<0.001
Female sexual function index III		27.04 ± 3.81		33.53 ± 1.55		<0.001
**(B) Study Group**						
**Female Sexual Function Index**		**Vulvovaginal Atrophy**				***p*** **(95% CI)**
		**Absent**	**Mild**	**Moderate**	**Severe**	
Visit 1		22.86 ± 6.27	-	14.40 ± 2.88	8.31 ± 2.58	<0.001
Visit 2		29.46 ± 6.20	19.61 ± 6.26	18.83 ± 6.35	9.3 *	0.001
Visit 3		31.40 ± 1.39	26.06 ± 3.47	-	-	<0.001
**(C) Study Group**						
**Female Sexual Function Index**		**DHEA Intravaginal Administration**				***p*** **(95% CI)**
		**No**		**Yes**		
Visit 2		25.09 ± 7.27		18.45 ± 5.62		0.006
Visit 3		31.50 ± 1.22		25.66 ± 3.22		<0.001
**(D) Study Group**						
**Visit no.**	**Vulvovaginal Atrophy, n (%)**	**DHEA Intravaginal Administration, n (%)**				***p*** **(95% CI)**
		**No**		**Yes**	**Total**	
2nd	Absent	7		0	7	<0.001
	Mild	3		23	26	
	Moderate	4		0	4	
	Severe	0		1	1	
	Total	14		24	38	
3rd	Absent	7		0	7	<0.001
	Mild	2		29	31	
	Total	9		29	38	

* means only one patient.

**Table 5:** Comparative evaluation of relationship/marital status through the study. Evolution of FSFI with age and couple/marital status in the study group.

**(A)**					
**Visit No.**	**Marital/Couple Status, n (%)**	**Group, n (%)**			***p*** **(95% CI)**
		**Study Group**	**Control Group**	**Total**	
1st	Single	28	15	43	0.005
	Relationship/Married	10	23	33	
	Total	38	38	76	
3rd	Single	24	7	31	<0.001
	Relationship/Married	14	31	45	
	Total	38	38	76	
**(B)**					
**Female Sexual Function Index**		**Marital/Couple Status of Study Group**			***p*** **(95% CI)**
		**Single**	**Relationship/Married**		
Visit 1		12.30 ± 6.71	16.31 ± 7.32		0.122
Visit 3		25.45 ± 3.70	29.77 ± 2.08		<0.001
**(C)**					
**Female Sexual Function Index**					
**Visit no.**	**Age**	**Marital/Couple Status of Study Group**			***p*** **(95% CI)**
		**Single**	**Relationship/Married**		
1st	20–27 years	15.58 ± 8.07	16.70 ± 13.43		0.866
	28 or older	9.46 ± 0.90	16.21 ± 6.56		0.004
3rd	20–27 years	26.39 ± 5.22	30.90 *		0.450
	28 or older	25.07 ± 0.72	29.68 ± 2.14		<0.001

* means only one patient.

## Data Availability

Data are contained within the article.
